# Isolation and Immunocharacterization of *Lactobacillus salivarius* from the Intestine of Wakame-Fed Pigs to Develop Novel “Immunosynbiotics”

**DOI:** 10.3390/microorganisms7060167

**Published:** 2019-06-06

**Authors:** Yuki Masumizu, Binghui Zhou, A.K.M. Humayun Kober, Md. Aminul Islam, Hikaru Iida, Wakako Ikeda-Ohtsubo, Yoshihito Suda, Leonardo Albarracin, Tomonori Nochi, Hisashi Aso, Keiichi Suzuki, Julio Villena, Haruki Kitazawa

**Affiliations:** 1 Food and Feed Immunology Group, Graduate School of Agricultural Science, Tohoku University, Sendai 980-0845, Japan; yuki.masumizu@bios.tohoku.ac.jp (Y.M.); binghui.zhou@bios.tohoku.ac.jp (B.Z.); humayuna2002@yahoo.com (A.K.M.H.K.); aminul.vmed@bau.edu.bd (M.A.I.); hikaru.iida@bios.tohoku.ac.jp (H.I.); wakako.ohtsubo@gmail.com (W.I.-O.); lalbarracin@herrera.unt.edu.ar (L.A.); ochi@m.tohoku.ac.jp (T.N.); 2 Livestock Immunology Unit, International Education and Research Center for Food Agricultural Immunology (CFAI), Graduate School of Agricultural Science, Tohoku University, Sendai 980-0845, Japan; asosan@tohoku.ac.jp; 3 Department of Dairy and Poultry Science, Chittagong Veterinary and Animal Sciences University, Chittagong 4225, Bangladesh; 4 Department of Medicine, Faculty of Veterinary Science, Bangladesh Agricultural University, Mymensingh-2202, Bangladesh; 5 Department of Food, Agriculture and Environment, Miyagi University, Sendai 980-0845, Japan; suda@myu.ac.jp; 6 Laboratory of Immunobiotechnology, Reference Centre for Lactobacilli (CERELA-CONICET), Tucuman 980-0845, Argentina; 7 Scientific Computing Laboratory, Computer Science Department, Faculty of Exact Sciences and Technology, National University of Tucuman, Tucuman 4000, Argentina; 8 Cell Biology Laboratory, Graduate School of Agricultural Science, Tohoku University, Sendai 980-0845, Japan; 9 Laboratory of Functional and Developmental Science of Livestock Production, Graduate School of Agricultural Science, Tohoku University, Sendai 980-0845, Japan; keiichi.suzuki@bios.tohoku.ac.jp

**Keywords:** wakame, *Lactobacillus salivarius*, gut microbiota, immunity, pigs

## Abstract

Emerging threats of antimicrobial resistance necessitate the exploration of effective alternatives for healthy livestock growth strategies. ‘Immunosynbiotics’, a combination of immunoregulatory probiotics and prebiotics with synergistic effects when used together in feed, would be one of the most promising candidates. Lactobacilli are normal residents of the gastrointestinal tract of pigs, and many of them are able to exert beneficial immunoregulatory properties. On the other hand, wakame (*Undaria pinnafida*), an edible seaweed, has the potential to be used as an immunoregulatory prebiotic when added to livestock feed. Therefore, in order to develop a novel immunosynbiotic, we isolated and characterized immunoregulatory lactobacilli with the ability to utilize wakame. Following a month-long in vivo wakame feeding trial in 8-week-old Landrace pigs (*n* = 6), sections of intestinal mucous membrane were processed for bacteriological culture and followed by identification of pure colonies by 16S rRNA sequence. Each isolate was characterized in vitro in terms of their ability to assimilate to the wakame and to differentially modulate the expression of interleukin-6 (IL-6) and interferon beta (IFN-β) in the porcine intestinal epithelial (PIE) cells triggered by Toll-like receptor (TLR)-4 and TLR-3 activation, respectively. We demonstrated that feeding wakame to pigs significantly increased the lactobacilli population in the small intestine. We established a wakame-component adjusted culture media that allowed the isolation and characterization of a total of 128 *Lactobacilli salivarius* colonies from the gut of wakame-fed pigs. Interestingly, several *L. salivarius* isolates showed both high wakame assimilation ability and immunomodulatory capacities. Among the wakame assimilating isolates, *L. salivarius* FFIG71 showed a significantly higher capacity to upregulate the IL-6 expression, and *L. salivarius* FFIG131 showed significantly higher capacity to upregulate the IFN-β expression; these could be used as immunobiotic strains in combination with wakame for the development of novel immunologically active feeds for pigs.

## 1. Introduction

The gastrointestinal (GI) tract of pigs is the residence of a dynamic microbial population that forms a complex ecosystem known as the microbiota, which has a symbiotic relationship with the host [1]. However, GI infections caused by pathogenic *Escherichia coli*, *Salmonella* spp., and *Clostridium perfringens* have been considered major health problems in weaning piglets, and have negative impacts on farm economics and food safety [2]. Antibiotics have widely been used to control GI infections in livestock including pigs; however, its indiscriminate usage might lead the development of antibiotic resistance, reduction of porcine beneficial gut microflora, and may have residual effects in humans [1,3]. In the race of increasing antibiotic usage—predicted to be increased by 67% from 2010 to 2030—antimicrobial drug resistance has emerged as a global problem for animal and human health, and scientists across the globe are vigorously searching for effective alternatives [4]. Prebiotics such as seaweed, and probiotic bacteria such as *Lactobacillus* spp. that have enhancing effects on the host´s immune function [5,6,7], in combination, could be potential candidates for antibiotic alternatives. The synergistic combination of immunoregulatory probiotics and prebiotics can be designated as Immunosynbiotics.

Among the seaweed, wakame (*Undaria pinnatifida*) is the most popular and economically important edible brown algae in Japan, Korea, and other Asian countries [8]. Seaweed contains various biologically active components that modulate the immune system function, including anti-inflammatory and antiviral activities [9,10]. One of the most abundant organic components in seaweed, following cellulose and hemicellulose, is alginate—the content of which is as high as 50% in wakame [11]. Only some bacteria have the capacity to utilize degraded polysaccharides [12,13]. In this study, we focused on wakame side products (e.g., root and stalk), to be used as prebiotic feed supplements as proper disposal of wakame wastes is crucial for the preservation of the coastal ecosystem and for the recycling of organic substances. Thus, it is essential to explore new technologies for the effective use of seaweed wastes along with the development of potential antibiotic alternatives for drug-free healthy livestock production. It is, however, noteworthy that seaweed, as a feed supplement, was previously reported to be beneficial for immune modulation in pigs [5]. Taken together, isolation and characterization of immunoregulatory probiotics capable of utilizing degraded polysaccharides might contribute to the development of immunosynbiotics through using wakame as a prebiotic supplement in the livestock feed.

Several recent studies have demonstrated that immunomodulatory probiotic microorganisms (immunobiotics) are an interesting alternative for improving the health of pigs. It was reported that ileal interleukin-10 (IL-10) expression showed a progressive increase in suckling piglets fed *Bifidobacterium longum* AH1206, which indicated the potential of this strain for modulating the inflammatory activity of the intestinal mucosa [14]. The probiotic strain *L. plantarum* CJLP243 has also been proposed as a potential antibiotic alternative to improve the health and production performance of weaning piglets because of its capacity to reduce the severity of enterotoxigenic *Escherichia coli* (ETEC)-induced diarrhea [15]. *L. delbrueckii* TUA4408L and its exopolysaccharides have shown the capability of increasing the expression of interferon beta (IFN-β), an antiviral cytokine, in porcine intestinal epithelial cells [16]. In addition, we previously established a porcine intestinal epithelial (PIE) cell line that demonstrated to be a useful in vitro tool for the screening and selection of immunomodulatory lactic acid bacteria (LAB) [17]. By using the PIE cell line, our group reported that immunobiotics could beneficially regulate the ETEC-induced intestinal epithelial cells’ immune response through the modulation of pro-inflammatory factors via regulation of Toll-like receptor (TLR) signaling [18,19]. Therefore, PIE cell-based screening of LAB isolates allows us to select the best immunobiotic candidates for their prospective use as antimicrobial substitutes in pigs.

We hypothesized that a synergistic combination of wakame and immunobiotic LAB, capable of metabolizing and growing in wakame, could be used as a highly efficient functional feed for antibiotic alternatives to enhance intestinal immunity and reduce the severity of infectious diarrhea in weaned piglets. Therefore, our main goal is to develop a novel immunosynbiotic comprising of the immunoregulatory prebiotic wakame and probiotic lactobacilli for pigs’ feed. To achieve this goal, we isolated and identified LAB strains from the gut of wakame-fed pigs, which were further evaluated in vitro for their capacity to utilize wakame and differentially modulate the host’s innate immune responses using PIE cells.

## 2. Materials and Methods

### 2.1. Wakame

By-products (stalk and root) of the wakame seaweed were collected from the area of the Miyagi Fishery Cooperatives Association, Omotehama beach, Toyohashi, Japan. The wakame wastes were washed thoroughly with water, dried, and milled to a fine powder by using a KDS device (Hitachi Zosen Corporation, Tokyo, Japan).

### 2.2. In Vivo Wakame Feeding Trial in Pigs

Six castrated 8-week-old Landrace pigs were housed in the Miyagi Prefecture Animal Industry Experiment Station (Miyagi, Japan). After one week of acclimatization with basic feed, pigs were divided into two groups: control and wakame-fed group. Commercial grower ration (Kitanihon Kumiai Feed Co, Ltd. Miyagi, Japan) was used as a basic diet. The wakame-fed group was fed with the experimental diet containing 1% wakame powder, while the control group was fed with the basic diet for the next four weeks. The amount of wakame in feed was determined based on the data from our previous study [20]. The experimental feed was supplied twice daily (morning and evening) with water supply ad libitum. Lighting and other management were carried out in accordance with standard husbandry practice. Body weight was measured at the beginning (9 weeks of age) and at the end (13 weeks of age) of feeding intervention. Average feed intake was estimated corresponding to weigh dates. The feed conversion ratio was determined by the ratio between body weight gain and average feed intake.

### 2.3. Preparation of Wakame Broth and Agar Media

Fine wakame powder was suspended in distilled water to make a 0.1% suspension. This suspension was autoclaved at 121 °C for 15 min and subjected for initial enzymatic hydrolysis. For hydrolysis, wakame broth was incubated at 37 °C for 24 h with the three enzymes: cellulase (0.05%) (Onoduka RS, Yakult Pharmaceutical Industry Co., Ltd., Tokyo, Japan), maserotime R-10 (0.05%) (Yakult Pharmaceutical Industry Co., Ltd., Tokyo, Japan), and alginic acid lyase (0.05%) (Nagase ChemteX Corporation, Tokyo, Japan). Hydrolyzed wakame broth was again autoclaved at 121 °C for 15 min, and followed with the separation of supernatant by centrifugation at 6000 rpm for 20 min. The supernatant containing wakame extract was further supplemented with yeast extract (0.1%) and NaCl (0.5%) and autoclaved at 121 °C for 15 min, and the wakame broth was thereby ready for use. In order to prepare the wakame agar medium, 1.5% agar was added to the wakame broth and supplemented with Bromocresol purple (BCP, 0.06%) as acid/alkali indicator.

### 2.4. Isolation and Characterization of Lactobacilli

For isolation of LAB, a section of the mucus membrane of the small intestine (jejunum, jejunum Peyer’s patches, ileum, and ileum Peyer’s patches) of pigs was collected. Pieces of mucosa were inoculated into 5 mL wakame broth and incubated at 37 °C for 24 h. For further screening of pure colonies, 100 μL of this fermentation broth was plated on modified 0.1% wakame agar medium supplemented with BCP (0.06%). After 96 h of incubation at 37 °C, the acid-producing yellow-colored colonies were grown and purified by repeated streaking on medium based on cultural characteristics (non-spore forming and non-hemolytic cultures), biochemical tests (catalase-negative) and staining properties (Gram-positive).

### 2.5. Morphological Characterization of Isolates

Gram staining and microscopic (Olympus IX70, Olympus Optical Co. Ltd., Tokyo, Japan) observation were initially used to characterize each isolated colony. In addition, the morphology of bacterial isolates was observed under a scanning electron microscope (SEM) (Hitachi, Tokyo, Japan). Briefly, the bacterial sample was serially diluted with Phosphate-Buffered Saline (PBS) to 10^1^–10^4^, 1 mL of the suspension was poured into a membrane filter (Polycarbonate Membrane, ADVANTEC, Tokyo, Japan) and suction filtered using a filter holder (Millipore®, Austin, TX, USA). The bacteria on the membrane filter were immobilized in 2% glutaraldehyde solution for 1 h at room temperature. The membrane was immersed in sterile water to remove excess glutaraldehyde. Thereafter, the membrane was soaked in 50, 60, 70, 80, 85, 90, 99, 100% (anhydrous) ethanol for 20 min at a time. Finally, the membrane was immersed in t-butanol, lyophilized, platinum palladium vapor deposited, and photographed with a scanning electron microscope (Hitachi, Tokyo, Japan).

### 2.6. Identification of LAB Isolates Using 16S rRNA Sequence

Genomic DNA was extracted from selected bacterial cultures using the DNeasy Kit (Qiagen, USA) following the manufacturer’s instructions. The DNA was quantified with a Nanodrop ND-2000 spectrophotometer (NanoDrop Technologies Wilimington, DE), and the integrity of DNA was determined by agarose gel electrophoresis. The DNA was stored at −20 °C until further analysis. The bacterial 16S rRNA gene was amplified by using forward primer 27f (5’ AGAGTTTGATCCTGGCTCAG 3’) and reverse primer 1492r (5’ GGTTACCTTGTTACGACTT 3’). The PCR was run for 3 min at 94 °C, and then 30 cycles at 94 °C for 30 s for denaturing; at 55 °C for 45 s for annealing; and 72 °C for 1 min for extension; followed by 7 min at 72 °C, and at 4 °C, using the Ex Taq system (Takara Bio Inc., Shiga, Japan). The resulting PCR products from each sample was quantified by capillary electrophoresis using an Agilent 2100 Bioanalyzer (Agilent Technologies, Carpinteria, CA, USA). After that, these PCR products were purified for gel electrophoresis. We used the Invitrogen E-Gel system for convenient loading, running, and elution of the size-selected DNA according to the manufacturer’s instructions. The library containing DNA fragments of about 1.5 kbp was amplified to a small extent to prepare for downstream emulsion PCR and sequencing steps. The sequencing of the purified PCR product was performed by BigDye Terminator v1.1 Cycle Sequencing Kit (Thermo, Co., Ltd., Foster City, CA, USA). The bacterial isolates were identified by Basic Local Alignment Search Tool (BLAST, http://blast.ncbi.nlm.nih.bov/Blast.cgi) through alignment of 16S rRNA gene sequences of bacterial isolates with the 16S rRNA sequence of a known species available in GenBank Database, and their closest relatives were determined. More than 99% of sequence similarities with a known species was used to consider the isolates to belong to a particular species.

### 2.7. Microbiota Analysis by Terminal Restriction Fragment Length Polymorphism (T-RFLP)

Terminal Restriction Fragment Length Polymorphism (T-RFLP)-based microbiota analysis was performed using the same intestinal tissue sections as used for LAB isolation. Genomic DNA was isolated using the DNeasy Kit (Qiagen, Germantown, MD, USA) following the manufacturer’s instructions. A pair of universal primers 35F (5’-CCTGCTCAGGATGAACG-3’) and reverse primer 1492r (5’-GGTTACCTTGTTACGACTT-3’) were used for PCR amplification. The purified PCR products were digested with 10 U/µL of AluI (TaKaRa) or MspI (TaKaRa) in a total volume of 10 µL at 37 °C for 1 h. The lengths of the terminal restriction fragments (T-RFs) were determined with the standard size marker GS500 ROX and 1000 ROX (Applied Biosystems, Foster City, CA, USA) using ABI PRISM^®^ 3130 genetic analyzer (Applied Biosystems) and GeneMapper software (Applied Biosystems). In order to confirm the increased Lactobacillus population in the porcine gut microbiota as a result of wakame feeding, we performed the sequence alignment of restricted cleavage site with the reference sequence of Lactobacillus salivarius by using MAFFT analysis software (http://mafft.cbrc.jp/alignment/software/). The peak of the cleavage site (CCGG) of Merozoite Surface Protein 1 (MSP I) (as around 56 to 573 bp) was counted from the first base of the 35 F primer sequence, where lactobacillus cluster was found.

### 2.8. In Vitro Evaluation of Wakame Assimilation Ability of LAB Isolates

The LAB isolates used for in vitro wakame assimilation study were first grown in deMann Rogosa and Sharpe (MRS) agar at 37 °C for 24 h. After that, 200 μL of this fermentation broth was subjected for sub-culture into the newly developed wakame-broth and incubate at 37 °C for 24 h. Starter culture was inoculated into 5 mL of three same wakame broth using L-shaped test tubes with optical density (OD) = 0.5 inoculated over time (measurement interval: 30 min, penetration rate: 60 rpm, total operation time: 24 h) (TVS026CA, Advantec, Tokyo, Japan). To test the acid survivability of LAB isolates, each sample in the fermentation broth was drawn at regular intervals of 6 h for estimation of viable cell counts and pH of the medium.

### 2.9. In Vitro Evaluation of Immunomodulatory Properties of LAB Isolates

A porcine intestinal epithelial (PIE) cell line originally established by our research group from intestinal epithelia of unsuckled neonatal swine [17], and was maintained in Dulbecco’s Modified Eagle Medium (DMEM) medium supplemented with fetal calf serum (10%), penicillin (100 U/mL), and streptomycin (100 mg/mL). The cultures were grown in a 250 mL flask at 37°C in a humidified atmosphere of 5% CO_2_. The cultures were passaged routinely after reaching the confluent of about 80–90% and used for continued experiments between the 25th and 35th passages.

The enterotoxigenic *Escherichia coli* (ETEC) 987P (O9:H−:987 pilus+: heat-stable enterotoxin+) was kindly provided by M. Nakazawa from the National Institute of Animal Health (Tsukuba, Japan). ETEC cells were grown in tryptic soy broth (TSB; Becton, Dickinson and Company, San Jose, CA, USA) for 24 h at 37 °C with continuous shaking. After overnight incubation, the subcultures of bacteria were centrifuged at 5000 × *g* for 10 min at 4 °C, washed with PBS and heat killed (100 °C, 30 min). All LAB isolates were grown in MRS medium (Difco, Detroit, MI, USA) for 16 h at 37 °C, washed with PBS, and heat killed (56 °C, 30 min). These bacterial samples were resuspended in DMEM, enumerated using a microscope and a Petroff-Hausser counting chamber and stored at −80 °C until use.

PIE cells were seeded at 3.0 × 10^4^ cells/mL in Type I collagen coated 12-well plates (SUMILON, Tokyo, Japan) and incubated at 37 °C with 5% CO_2_ for 3 days. On the third day of culture, the LAB isolates were added (100 MOI, multiplicity of infection) and stimulated for 48 h. After washing the wells with PBS, 3.4 μL (3.0 × 10^7^ cells/mL) of ETEC or 5 μL (100 ng/mL) of poly(I:C) (Sigma-Aldrich, St. Louis, MI, USA) was added and maintained for 12 h. Finally, after the PBS wash, 500 μL TRIzol reagent was added to each well and transferred to a 1.5 mL micro tube for RNA extraction according to the manufacturer’s instruction.

### 2.10. Quantitative Real-Time PCR

Total RNA samples were extracted from treated and control PIE cells using TRIzol reagent (Invitrogen, Carlsbad, CA, USA) and treated with gDNA Wipeout Buffer (Qiagen, Tokyo, Japan). RNA was reverse transcribed using a Quantitect reverse transcription (RT) kit (Qiagen, Tokyo, Japan) according to the manufacturer’s recommendations. The synthesized cDNA was then used for quantitative PCR analysis using Platinum SYBR green qPCR SuperMix UDG with ROX (Invitrogen, Carlsbad, CA, USA) on 7300 real-time PCR system (Applied Biosystems, Warrigton, United Kingdom). The primers used for the analysis of IL-6, IFN-β, and β-actin were described in previous publications [21,22]. PCR cycling conditions were 5 min at 50 °C, followed by 5 min at 95 °C, and then 40 cycles of 15 s at 95 °C, 30 s at 60 °C, and 30 s at 72 °C. Expression of β-actin in each sample was assessed, and the β-actin data were used as an internal control to normalize differences between samples and calculate relative expression levels. Samples were run in triplicate for each experimental condition, and mean values were used to calculate statistics.

### 2.11. Discriminant Analyses

In order to characterize the bacteria based on gene expression profiles, the discriminant function analysis was carried out, and its significance of a discriminant function with explained variables of cytokine gene expressions was examined by hypothesizing that 4 or 5 kinds of bacteria groups have each similar immunological function. The weighting of explained variables was performed to distinguish the most significant 4 or 5 bacteria groups, and then calculated discriminant scores were used as an index to characterize each significant group of bacteria based on cytokine expression. The distribution of their scores was standardized and displayed by a 2-dimensional plot. A discriminant function to calculate its scores was as follow.

$$y=a_{1}x_{1}+a_{2}x_{2}+a_{3}x_{3}+\cdots \cdots \cdots +a_{n}x_{n}$$

where, y are discriminant scores, $a_{1},\cdots \cdots ,a_{n}$ are weights of cytokine gene expressions and $x_{1},\cdots \cdots \cdots ,x_{n}$ are cytokine gene expressions.

### 2.12. Statistical Analysis

The statistical analyses were performed by using BellCurve for Excel (Social Survey Research Information Co., Ltd, Tokyo, Japan). Data were presented as the mean ± SD. Differences between the control and were assessed using one-way ANOVA followed by the independent two-tailed *t*-test. Differences were considered as significant when *p* < 0.05.

### 2.13. Ethics Statements

The experimental protocol strictly followed the animal handling guidelines for animal experiments of Agriculture and Environmental Science, Miyagi Prefecture Animal Industry Experiment Station, Iwadeyama-machi, Japan. The present experiment was approved by the Laboratory health and safety Committee of Miyagi University with the permission No. 2015-17 dated on 01 April 2015 and No. 2017-26 dated on 31 March 2017.

## 3. Results

### 3.1. Effect of Wakame Feeding on Pigs

**Figure 1:**
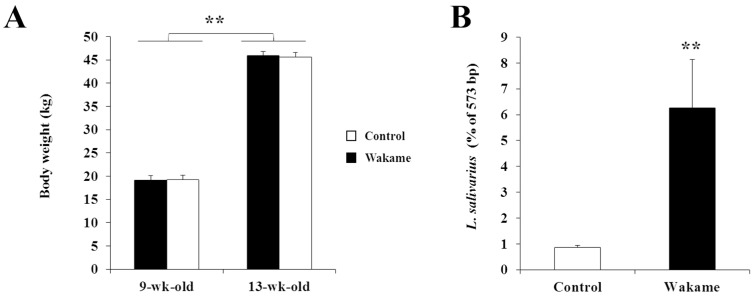
Effect of wakame feeding on body weight gain and gut microbiota of pigs. (**A**) Body weight of pigs at the beginning (9 weeks-old pigs) and the end (13 weeks-old pigs) of dietary intervention. (**B**) Proportion of *Lactobacillus salivarius* population in the porcine gut microbiota estimated by terminal restriction fragment length polymorphism (T-RFLP) method followed by sequence (573 bp) alignment using MAFFT tool. Asterisks (**) indicate statistical differences between wakame-fed and control groups with a significant level of *p* < 0.01.

The analysis of data obtained from the live animal wakame feeding trail showed that wakame supplementation did not significantly influence the usual growth pattern of pigs. The average body weights of wakame-fed and control pigs were similar in both time points evaluated: at the beginning (at 9th weeks of age) and at the end of feeding trial (at 13 weeks of age), indicating that the rate of body weight gain was similar in two groups (Figure 1A). In addition, no clinical abnormalities were observed in both groups, indicating the absence of toxic properties in the wakame.

In order to explore the effects of wakame feeding on the porcine gut microbiota, we performed microbiota profiling in the intestinal mucosa from both wakame-fed and control pigs. We used the T-RFLP analysis method for characterizing the composition of gut microbiota. Results revealed that *Lactobacillus salivarius* represented about 6.3% of gut microbiota community in the wakame-fed group, while this proportion corresponded to 0.85% in the control group (Figure 1B). These results indicated that wakame feeding led a higher population of *L. salivarius* in the gut microbiota of pigs.

### 3.2. Development of Wakame Broth and Wakame Agar Media

For initial growth and propagation of LAB, we prepared a liquid medium containing dissolved wakame powder in water. Dissolution of powder was performed by treating the wakame with different combinations of three hydrolytic enzymes: alginate lyase, cellulase, and maserotime R-10. Enzymatic activity and bacterial growth were evaluated by measuring the turbidity and transmissivity of the broth over time. Results indicated that the lowest turbidity (OD = 0.29) and the maximum transmissivity (50%) was obtained when the broth was treated with the three enzymes as compared with the single or dual treatments (Supplementary Table S1). Therefore, the combination of these three enzymes for dissolving wakame powder was used in further experiments.

To optimize the dose of enzymatic treatment, we treated the solution of wakame powder with different concentrations of the three enzymes and measured the turbidity and transmissivity of the solution. The turbidity was decreased, and the transmissivity was enhanced when concentrations were increased from 1 mg/10 mL up to 5 mg/10 mL of each enzyme (Supplementary Table S2). However, a further increment of enzymes doses from 6 mg/10 mL to 10 mg/10 mL did not induce significant differences in decreasing turbidity and increasing transmittance (Supplementary Table S2). Therefore, a 0.1% solution of wakame power was dissolved with three enzymes in a concentration of 5 mg/10 mL to prepare the wakame broth.

**Figure 2:**
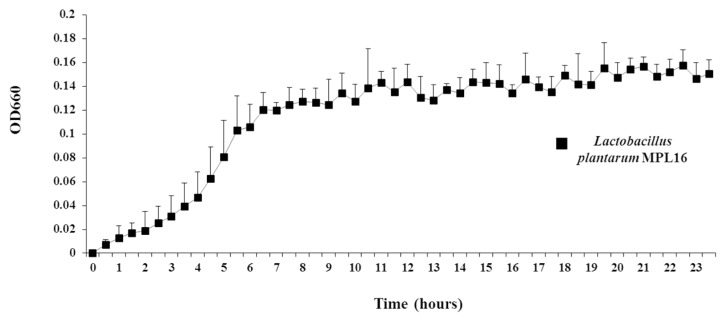
Evaluation of newly developed wakame medium by estimating the optical density (OD) of broth following the culture of *Lactobacillus plantarum* MPL16. The OD of wakame-broth used was measured progressively at every half an hour incubation at 37 °C for 24 h.

For the isolation of individual wakame-assimilating LAB colonies, we prepared a wakame agar medium by adding 1.5% agar into the wakame broth. In order to optimize the nutritional composition of the wakame agar, we performed growing experiments with a known LAB wakame-assimilating isolate, *Lactobacillus plantarum* MPL16 [23]. We observed no single colonies in the wakame agar (only 1.5% agar added to the 0.1% wakame solution) medium even after 96 h of inoculation of *L. plantarum* MPL16 (Supplementary Figure S1A). Therefore, wakame agar medium was enriched by adding 0.5% NaCl and/or 0.1% yeast extract to promote growth and proliferation of lactobacilli. Then, colony formation was clearly visible following inoculation of *L. plantarum* MPL16 in to the enriched wakame agar medium after 96 h of incubation at 37 °C (Supplementary Figures S1B–D). The scanning electron microscopy, Gram staining (Supplementary Figure S2A), and the OD estimates of wakame broth (Figure 2) cultured with *L. plantarum* MPL16 also confirmed its growth in the wakame-based medium. By this way—1.5% agar added to the 0.1% wakame solution containing 0.5% NaCl, 0.1% yeast extract, and 0.06% Bromocresol purple (as pH indicator)—the component adjusted wakame agar medium was optimized for selective isolation of wakame fermenting LAB.

### 3.3. Isolation and Identification of Porcine Wakame-Assimilating Lactobacilli

Using the newly developed wakame broth and agar media, we isolated LAB from the intestinal mucosa of both wakame-fed pigs and control pigs. Propagation of LAB in the wakame medium was determined by the color change pattern of agar media as indicated by BCP. Following inoculation, there was a distinct pattern of color change between the agar media plated with a sample obtained from wakame-fed pigs and the sample from control pigs. After 24 h of incubation with bacteriological samples from control pigs, the color of agar medium turned from purple to dark blue, indicating no growth of lactobacilli. On the other hand, the color of agar plates containing samples of wakame-fed pigs changed from purple to dark blue after 24 h incubation. However, after a subsequent 96 h of cultivation, there were several plates in which the color of the BCP indicator changed to yellow around the colonies. Therefore, the production of acidic compounds and bacterial growth was clearly noticed in samples obtained from wakame-fed pigs. A total of 128 pure bacterial colonies were obtained from the wakame-fed group. The morphological features of isolated LAB were analyzed. Scanning electron microscopy and Gram staining (Supplementary Figure S2B,C) revealed that all the isolates were rod-shaped and Gram-positive bacteria. In addition, all these 128 colonies were subjected to 16S rRNA sequencing individually. The BLAST analyses of 16S rRNA sequence identified that all isolates belonged to *Lactobacillus salivarius* species which were isolated from the intestine of wakame-fed pigs.

### 3.4. Wakame Fermentation Ability of LAB Isolates

**Figure 3:**
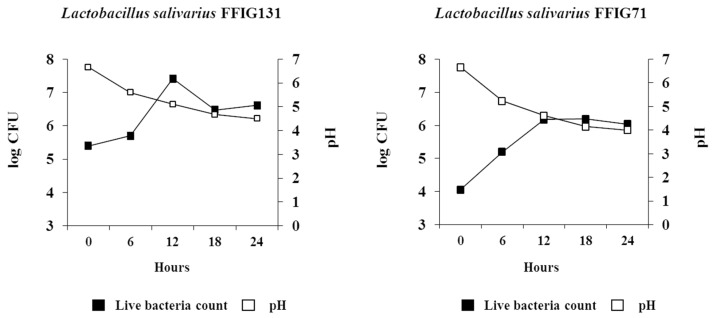
Change of pH and viable bacterial counts of *Lactobacillus salivarius* wakame assimilating isolates in the newly developed wakame-component adjusted media. *Lactobacillus salivarius* FFIG71 is the strongest wakame-utilizing and anti-inflammatory isolate (based on OD and IL-6 expression values presented in Table S3). *Lactobacillus salivarius* FFIG131 is the strongest wakame utilizing and antiviral isolate (based on OD and IFN-β expression values presented in Table S3). Both isolates were cultured in newly developed wakame broth for 24 h and pH of the medium and viable bacterial count was estimated to evaluate the bacterial growth on the medium.

The LAB colonies were evaluated individually for their ability to assimilate wakame in vitro. The turbidity was measured by optical density (OD) at every 30 minutes over a 24 h culture period, and the wakame assimilation ability of growing bacteria was evaluated by the increased turbidity rate of culture broth. The wakame assimilation of *L. salivarius* isolates resulted in OD value ranges from 0.01 to 0.3 (supplementary Table S3). These results indicated that the wakame assimilation ability greatly differs among the different isolated colonies. For further confirmation of wakame assimilation by the isolates, we estimated the pH of wakame broth and the viable bacterial counts in the wakame agar over a period of 24 h growth. The strains with high turbidity (OD) in the wakame broth also showed an increasing growth tendency over the 24 h of culturing in the wakame agar medium with a peak proliferation to 10^7.5^ cfu/mL at 12 h of incubation. A decreasing pH trend in the media was observed over time in this group of bacteria (Figure 3). On the other hand, for the isolates with low OD values in the wakame broth, their growth tended to show up until 12 h after culture in the wakame agar, but their viable count stopped raising at 10^6^ cfu/mL, though a decreasing trend of pH over time was confirmed (Figure 3). These results indicate that *L. salivarius* colonies isolated from the intestine of wakame-fed pigs also have the potential of assimilating wakame powder used in the culture medium.

### 3.5. Immunoregulatory Properties of LAB Isolates

**Figure 4:**
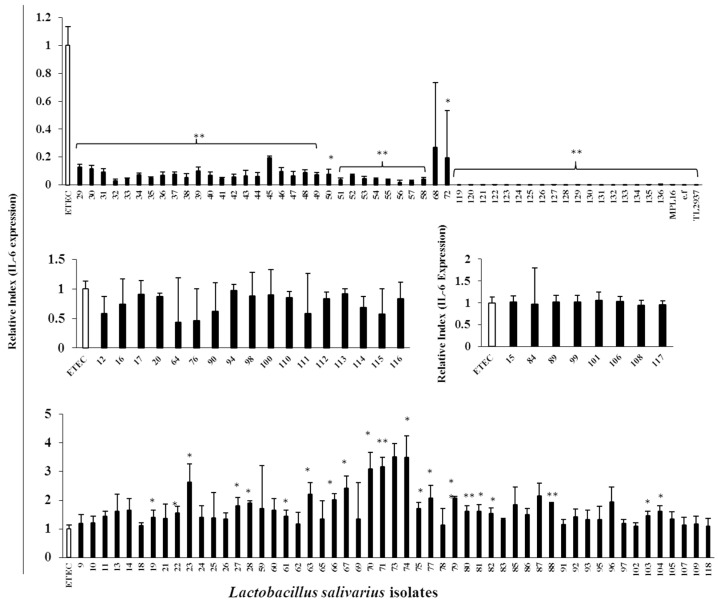
Immunoregulatory properties of *Lactobacillus salivarius* isolates. Porcine Intestinal Epithelial (PIE) cells were pre-stimulated with each isolate for 48 h followed by Enterotoxigenic *E. coli* (ETEC) challenge for 12 h, and then quantification of IL-6 mRNA expression by qRT-PCR was done. The Y-axis represents the relative index of IL-6 expression, and X-axis represents the individual isolates of *L. salivarius* indicated by the chronological numbers (FFIG9-FFIG136). MPL16 and TL2937, two known *Lactobacillus* strains, were used as positive controls. Results displayed in the bar graphs represent the mean ± SD of three independent experiments performed in triplicates. Statistical differences between ETEC-challenged control PIE cells and bacterial prestimulated PIE cells followed by ETEC challenge are indicated with asterisks (*) *p* < 0.05 and (**) *p* < 0.01.

The potential immunomodulatory capacities of all the 128 LAB isolated colonies were evaluated in a PIE cell model. For this purpose, PIE cells were pre-stimulated with each isolate for 48 h, followed by ETEC challenge for 12 h for TLR4 stimulation [18]. Then, the expression of IL-6 was measured. Taking into consideration the changes in the levels of IL-6 expression, lactobacilli isolates were divided into four groups: 50 *L. salivarius* isolates with the ability to strongly decrease IL-6, 15 *L. salivarius* isolates that moderately decreased this cytokine, 55 strains that increased IL-6, and 8 isolates with no effect on IL-6 expression in PIE cells after ETEC challenge (Figure 4).

**Figure 5:**
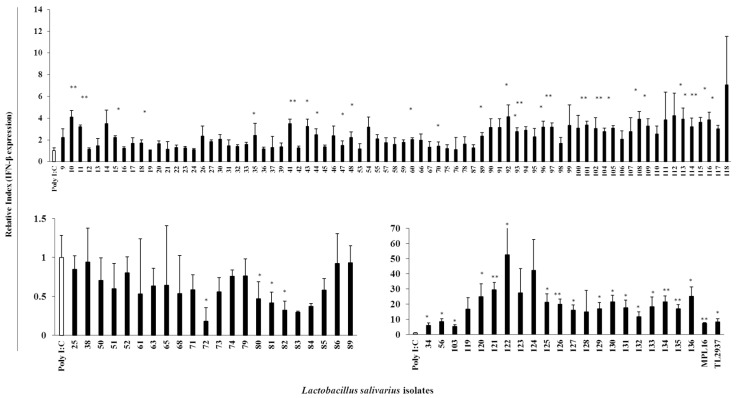
Immunoregulatory properties of *Lactobacillus salivarius* isolates. PIE cells were pre-stimulated with each isolate for 48 h followed by Poly(I:C) challenge for 12 h, and quantification of IFN-β mRNA expression by qRT-PCR was done. The Y-axis represent the relative index of IFN-β expression, and X-axis represent the individual isolates of *L. salivarius* indicated by chronological number (FFIG9-FFIG136). MPL16 and TL2937, two known *Lactobacillus* strains, were used as positive controls. Results displayed in the bar graphs represent the mean ± SD of three independent experiments performed in triplicates. Statistical differences between ETEC-challenged control PIE cells and bacterial prestimulated PIE cells followed by ETEC challenge are indicated with asterisks (*) *p* < 0.05 and (**) *p* < 0.01.

In addition, the ability of LAB isolates to modulate TLR3-mediated immune responses in PIE cells was evaluated by measuring IFN-β expression after poly(I:C) challenge. Result revealed that 23 *L. salivarius* isolates increased this type I interferon while 9 isolates had no effect. Moreover, 22 and 60 isolates strongly and moderately reduced IFN-β expression in PIE cells after poly(I:C) challenge, respectively (Figure 5).

### 3.6. Relationship Between Wakame Assimilation and Immunoregulatory Effects of LAB Isolates

**Figure 6:**
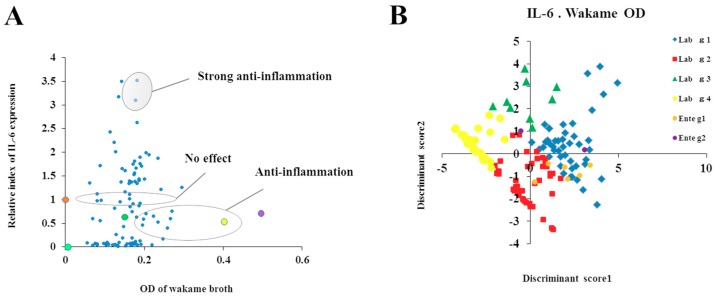
Discriminant analysis of wakame assimilation and immunoregulatory activity of isolates. (**A**) Relationship between IL-6 expression modulation and wakame assimilation (OD) of *Lactobacillus salivarius* isolates. (**B**) Discrimination of conveniently categorized groups of bacterial isolates estimated by discriminant function analysis based on IL-6 expression and wakame assimilation.

**Figure 7:**
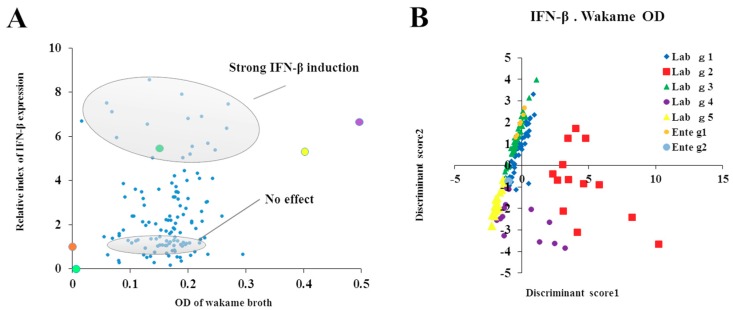
Discriminant analysis of wakame assimilation and immunoregulatory activity of isolates. (**A**) Relationship between IFN-β expression modulation and wakame assimilation (OD) of *Lactobacillus salivarius* isolates. (**B**) Discrimination of conveniently categorized groups of bacterial isolates estimated by discriminant function analysis based on IFN-β expression and wakame assimilation.

Discriminant function analysis was performed to further categorize the LAB isolates based on the relationship between the wakame assimilation and the immunomodulatory capacity of each isolate (supplementary Table S3). For this, IL-6 expression was considered as the “anti-inflammatory phenotype”, IFN-β expression was considered as the “IFN-β expression phenotype”, and OD values of wakame broth after 24 h incubation with LAB isolates were considered as the “assimilation property phenotype”. LAB isolates were categorized into four major groups based on OD value and IL-6 expression and; five major groups based on OD value and IFN-β expression (Table 1). The 2-D plots illustrated the relative value of IL-6 or IFN-β on the vertical axis and the final OD value after 24 h at the time of cultivation in wakame culture medium on the horizontal axis (Figure 6 and Figure 7). Based on the regulatory potential of inflammation, *L. salivarius* isolates were divided into three groups: anti-inflammatory strains, pro-inflammatory bacteria, and isolates with no effects (Figure 6A). Based on the regulatory potential of IFN-β expression, *L. salivarius* isolates were grouped into three: lactobacilli that increased IFN-β expression, bacteria that decreased IFN-β expression, and isolates with no effects (Figure 7A). Based on discriminant function analysis, the relationship between IL-6 and wakame-assimilation could be significantly characterized into 4 groups (*p* < 0.01) (Figure 6B). On the other hand, although IFN-β can be characterized similarly since the population is biased on the Y axis, it was suggested that further discrimination would be enhanced by adding more relevant factors in future (*p* < 0.01) (Figure 7B). Importantly, among the wakame assimilating isolates, *L. salivarius* FFIG71 showed a significantly higher capacity to upregulate IL-6 expression, and *L. salivarius* FFIG131 showed a significantly higher capacity to upregulate IFN-β expression. 

## 4. Discussion

The population of gut microbiota, in particular, lactobacilli, plays key roles in maintaining the nutritional, physiological, and immunological functions of pigs [24,25]. It was proposed that changes in the diet composition are a sustainable option to improve microbial diversity and beneficial microbes [1]. In this sense, Liu et al. [25] investigated the effects of dietary fibers on the growth and performance of piglets as well as on the composition of their gut microbiota. The work reported that dietary fibers could alter the composition of gut microbiota, thereby reducing the incidence of diarrhea in piglets. Other studies have also confirmed that dietary fibers are able to contribute to the development, health, and beneficial micro-ecological balance of piglet’s gut [26]. Interestingly, it was reported that feeding pigs with a diet supplemented with the cultivated red seaweed *Chondrus crispus* beneficially affected the host immunity and the colonic microbiota [27]. Similarly, in this study, we reported for the first time that wakame feeding to pigs resulted in a significant change in gut microbiota. Although pigs’ body weight gain was not affected by wakame feeding, the composition of gut microbiota was significantly changed with a high abundance of *Lactobacillus* spp. The modification of porcine gut microbiota may be the result of enrichment of wakame derived fermentable substrates in pig’s intestine. Thus, our results allow the speculation that wakame feeding leads to changes in pig GI environments through the release of carbonaceous products derived from seaweed, resulting in changes in the expression of loci associated with seaweed assimilation in residential lactobacilli. In line with this hypothesis, some works have demonstrated that alterations in gut microbial diversity are attributed to the ability of bacterial strains to adapt or not to dietary changes [28]. An increased number of *Bifidobacterium breve* has been observed in rats after feeding with brown algae due to its prebiotic effects [27]. Similarly, a higher number of *Bifidobacterium* species was noticed in rats after feeding high indigestible raffinose [29]. It should be noted that another possibility to explain the changes in pigs´ microbiota is the introduction of bacterial groups into the gut directly from the surfaces of the seaweed itself. The origin of the increased populations of *Lactobacillus* ssp. in general, and *L. salivarius* in particular, in the gut of pigs after wakame administration deserves further investigation.

Another point of importance in this work is that we have successfully developed a wakame-component adjusted culture media for selective isolation of wakame-fermenting LAB as well as for the *in vitro* evaluation of their wakame-assimilation properties. For the preparation of the wakame-based culture medium, wakame powder was required to be dissolved by enzymatic treatment. This treatment was necessary, taking into consideration that alginic acid of wakame forms sodium alginate in the presence of saline sea water and that this viscous sodium alginate remains in the wakame extract. In fact, the wakame roots collected from Miyagi prefecture contained 23.26% of sodium alginate, which remained about 2.5% in the grinned seaweed powder. Then, we employed three selected catalytic enzymes, namely alginate lyase, cellulase onozuka RS, and maserotime R-10, for the enzymatic hydrolysis of wakame powder. This selection was performed by considering other studies in which the dissolution of seaweed powder was successful [30,31,32]. The enzymatic treatment was also necessary for the growth promotion of wakame assimilating lactobacilli. In the gut environment, it is probable that some other members of gut microbiota digest the wakame crude powder to some extent, and the lactobacilli population could then utilize the partially digested wakame. For this reason, in the selective wakame bacterial culture medium, the enzymatic treatments were required to allow the growing lactobacilli in it.

Moreover, in order to facilitate the isolation of single pure colonies of wakame assimilating bacteria, we also developed a wakame agar media by adding to the broth 1.5% of agar. However, it should be mentioned that the growth of wakame assimilating strains such as *L. plantarum* MPL16 was not satisfactory in the basic wakame agar medium when compared to commercial mediums for lactobacilli. These results indicated that although there are components required for the growth of LAB in enzyme treated aqueous solution of wakame powder, bacterial proliferation may require additives other than the sugar source [33]. Therefore, it was necessary to optimize the composition of the agar medium in order to promote the growth of bacteria. Shobharani et al. [33] reported that adequate growth of *L. plantarum* could be ensured by using 2.5% or less of NaCl in the medium. On the other hand, commercially available culture mediums such as MRS, BRIGGS, and M17 agar that have long been used for selective isolation of *Lactobacillus* [34] contain yeast extracts. For example, MRS medium contains 0.4% yeast extract and 0.5% sodium acetate [34]. Considering those facts, we evaluated whether the addition of NaCl and yeast extract as supplements for the wakame agar medium was able to promote the growth of LAB. It was found that the addition of 0.1% yeast extract and 0.5% NaCl further promoted the proliferation of *L. plantarum* MPL16 in the wakame agar medium.

Taking into consideration that the porcine intestinal bacterial populations were changed due to wakame feeding and that it was previously reported that wakame administration was able to modulate the pigs´ immune system [5]; we hypothesized that some bacterial strains would be responsible for the immunomodulatory effect of wakame. Then, we aimed to prepare a *Lactobacillus* spp. library from wakame-fed pigs and investigate their immunomodulatory capacities. We cultured the intestinal section of wakame fed pigs in a customized wakame-based agar medium that enabled us to isolate 128 pure bacterial colonies, which were subsequently identified as belonging to the *L. salivarius* species as per their 16S rRNA sequence homology. These findings suggested that the increased *Lactobacillus* population in the porcine gut induced by wakame feeding could be attributed mainly to the increase of the *L. salivarius* population. The high growth performance of *L. salivarius* colonies isolated from the intestine of wakame-fed pigs, in the wakame-based culture medium confirmed their high potential to ferment the edible algae.

For the evaluation of the immunomodulatory capacities of *L. salivarius* isolates, we performed studies in PIE cells prestimulated individually with each of the 128 isolates followed by challenges with TLR4- or TLR3-agonists. As expected, isolate-dependent abilities to modulate immune responses triggered by TLRs activation in PIE cells were found. Some *L. salivarius* isolates showed a high wakame assimilation ability and immunomodulatory capacity in PIE cells. Therefore, we proposed those isolates as potential immunobiotic candidates to beneficially modulate gastrointestinal immunity in the porcine host. Although the existence of a correlation between the seaweed assimilation and immunomodulatory ability was indicated from discriminant analysis, the causal relationships thereof can be confirmed by comparative genomes of related strains and host immunotranscriptional responses using PIE cells as well as in vivo settings.

It should be considered that analysis of the 16S rRNA region performed here does not allow us to state whether the 128 isolates were unique strains. However, discriminant analysis identified the isolates with distinctive phenotypes (upregulation of IL-6 and IFN-β expression) are not likely be of the same clones. To this end, further studies regarding the clonality of the 128 *L. salivarius* isolates such as the determination of whole genome sequences or detailed evaluation of their metabolic activities are necessary to estimate the number of different *L. salivarius* strains that co-exist in the pig intestine. Those studies are also important to determine the numbers of unique strains that belong to the major phenotypic groups found in this work when considering the wakame assimilating abilities and the immunomodulatory functions. In this regard, comparative genomic studies between the wakame assimilating isolates such as *L. salivarius* FFIG71 and *L. salivarius* FFIG131—which have the ability to upregulate the IL-6 and IFN-β expression in PIE cells, respectively—would be of great value to advance in the characterization of unique *L. salivarius* strains for the development of immunosynbiotics.

## 5. Conclusions

In this work we: a) demonstrated for the first time that wakame feeding to pigs significantly changes microbiota composition by increasing bacterial species such as lactobacilli that could confer beneficial effects to the porcine host, b) established wakame-component adjusted culture media that allowed the selection and characterization of promising LAB isolates with high potential to be used in the development of seaweed fermented feeds, and c), selected from the wakame-assimilating LAB isolates those with the ability to differentially modulate innate immune responses triggered by TLR4 and TLR3 activation in PIE cells. Some *L. salivarius* isolates showed a high wakame assimilation ability and remarkable immunomodulatory capacity. Therefore, these results suggest that those *L. salivarius* isolates (e.g., *L. salivarius* FFIG71 and *L. salivarius* FFIG131) could be used as immunobiotic candidates for the development of new immunologically active feeds, which might contribute to improving immune health status in the porcine host. However, the precise molecular mechanisms behind the immunomodulatory activities and the metabolic pathways related to the seaweed assimilation of immunomodulatory *L. salivarius* isolates remain to be elucidated. In addition, the whole genome sequence analysis of the *L. salivarius* isolates with strong immunomodulatory and wakame-assimilation properties would enable insight into this genotype-phenotype relationship; these are interesting topics for future studies in order to develop effective immunosynbiotics.

## Figures and Tables

**Table 1:** Categorization of the isolates based on wakame assimilation (OD) and immunomodulatory (Relative index of IL-6 and IFN-β expression) properties.

Phenotype	Group	Ranges of OD and RI Estimates *	Combined Properties
IL-6	g1	OD ≥ 0.1; RI ≥ 1	Isolates able to utilize wakame and strongly induce anti-inflammation
	g2	OD ≥ 0.1; RI < 1	Isolates able to utilize wakame and induce anti-inflammation
	g3	OD < 0.1; RI ≥ 1	Isolates cannot utilize wakame but strongly induce anti-inflammation
	g4	OD < 0.1; RI < 1	Isolates cannot utilize wakame but induce anti-inflammation
IFN-β	g1	OD > 0.1; 10 ≥ RI ≥ 2	Isolates able to utilize wakame and induce IFN-β expression
	g2	OD > 0.1; RI ≥ 10	Isolates able to utilize wakame and induce strong IFN-β expression
	g3	OD > 0.1; RI ≤ 1	Isolates able to utilize wakame and induce anti-inflammation
	g4	OD ≤ 0.1; RI ≥ 2	Isolates cannot utilize wakame but induce IFN-β expression
	g5	OD ≤ 0.1; RI ≤ 1	Isolates cannot utilize wakame but induce anti-inflammation

* OD, Optical Density; RI, Relative Index.

## Supplementary Materials

The following are available online at https://www.mdpi.com/2076-2607/7/6/167/s1. Figure S1. Development and optimization of wakame-based agar medium. (A) Only 0.1% wakame medium. (B) 0.1% wakame medium with 0.1% yeast extract. (C) 0.1% wakame medium with 0.5% sodium chloride (NaCl). (D) 0.1% wakame medium with 0.1% yeast extract and 0.5% NaCl. In all cases, 1.5% agar was added. Agar media were inoculated with *Lactobacillus plantarum* MPL16 and cultured for 96 h and the colony growth demonstrated by red circles. Figure S2. Morphological characteristics of lactobacillus isolates by scanning electron microscopy (SEM) and Gram staining. (A) *Lactobacillus plantarum* MPL16, a known wakame assimilating strain, at magnifying power 30 kV 8.9 mm × 6.00 k, scale bar 5 μm; magnifying power 30 kV 8.8 mm × 18.0 k, scale bar 3 μm; and Gram staining, scale bar 10 µm. (B) *Lactobacillus salivarius* FFIG71, a wakame-utilizing and anti-inflammatory isolate (based on OD and IL-6 expression presented in Table S3) at magnifying power 30 kV 8.9 mm × 18.0 k scale bar 5 μm; 30 kV 9.0 mm × 6.00 k scale bar 3 μm, and Gram staining image. (C). *Lactobacillus salivarius* FFIG131, a strongest wakame utilizing and antiviral isolate (based on OD and IFN-β expression presented in Table S3) at magnifying power 30 kV 8.9 mm × 18.0 k scale bar 5 μm; 30 kV 9.0 mm × 6.00 k scale bar 3 μm; and Gram staining image. Table S1. Dissolution of wakame powder by different combinations of enzymatic treatments. Table S2. Optimization of the doses of enzymatic treatments used for hydrolysis of wakame powder. Table S3. The immunomodulatory abilities and wakame-assimilation abilities of all 128 *L. salivarius* isolates. The IL6 and IFNβ mRNA expressions in porcine intestinal epithelial cells represents the immunomodulatory abilities and the optical density (OD) of wakame broth containing lactobacilli represent the wakame assimilation abilities of the isolates.
