# Supplementary material for: Isolation and Immunocharacterization of Lactobacillus salivarius from the Intestine of Wakame-Fed Pigs to Develop Novel “Immunosynbiotics”
Source: Microorganisms. 2019 Jun 6;7(6):167. doi: 10.3390/microorganisms7060167 (PMC6617407; doi:10.3390/microorganisms7060167)
Supplement: Supplementary file 1 [file microorganisms-07-00167-s001.zip › microorganisms-470891-proofreading-supplementary/Supplementary files/Supplementary Tables S1 and S2.docx]

**Supplementary Tables**

**Table S1.** Dissolution of wakame powder by different combinations of enzymatic treatments.

|  |  | **Treatment Combinations** (+ indicates the adding of corresponding enzymes) | | | | | | | |
| --- | --- | --- | --- | --- | --- | --- | --- | --- | --- |
| **Enzymatic Treatments** | **Alginic Acid Lyase** |  | + | + | + |  | + |  |  |
|  | **Cellulase Onoduka RS** |  | + | + |  | + |  | + |  |
|  | **Maserotime R-10** |  | + |  | + | + |  |  | + |
| **Results** | **Turbidity (OD660)** | 0.43 | 0.29 | 0.34 | 0.37 | 0.45 | 0.31 | 0.43 | 0.43 |
|  | **Transmissivity (%)** | 37.6 | 50 | 44 | 41.8 | 33.6 | 47.5 | 36 | 38.8 |
|  | **Broth Appearance** | 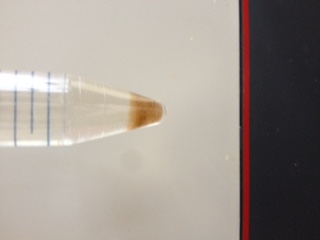 | 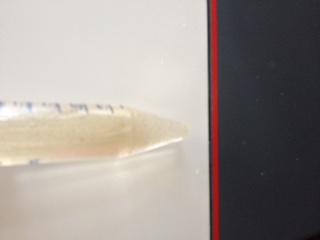 | 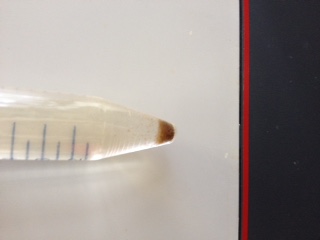 | 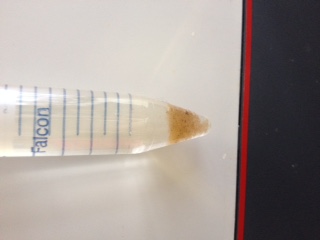 | 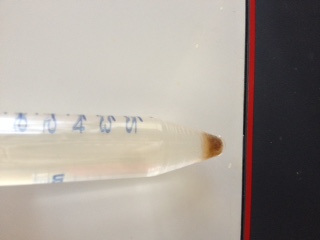 | 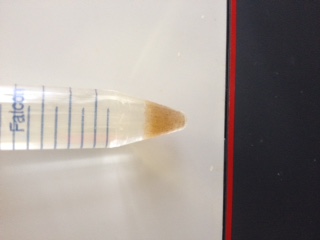 | 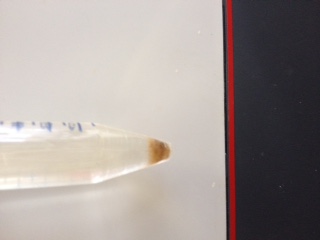 | 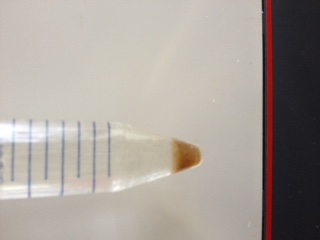 |

**Table S2.** Optimization of the doses of enzymatic treatments used for dissolution of wakame powder.

|  | **Concentration of Three Enzymes Combined Used** (mg/10 mL of broth) | | | | | | | | | | |
| --- | --- | --- | --- | --- | --- | --- | --- | --- | --- | --- | --- |
|  | **0** | **1** | **2** | **3** | **4** | **5** | **6** | **7** | **8** | **9** | **10** |
| **Turbidity (OD660)** | 0.81 | 0.526 | 0.491 | 0.492 | 0.499 | 0.437 | 0.468 | 0.524 | 0.555 | 0.577 | 0.6 |
| **Transmissivity (%)** | 15.7 | 29.5 | 32.2 | 32.1 | 32.2 | 37.2 | 34 | 29.6 | 28.1 | 26.2 | 25 |
