# Supplementary figures and images for: Isolation and Immunocharacterization of Lactobacillus salivarius from the Intestine of Wakame-Fed Pigs to Develop Novel “Immunosynbiotics”
Source: Microorganisms. 2019 Jun 6;7(6):167. doi: 10.3390/microorganisms7060167 (PMC6617407; doi:10.3390/microorganisms7060167)

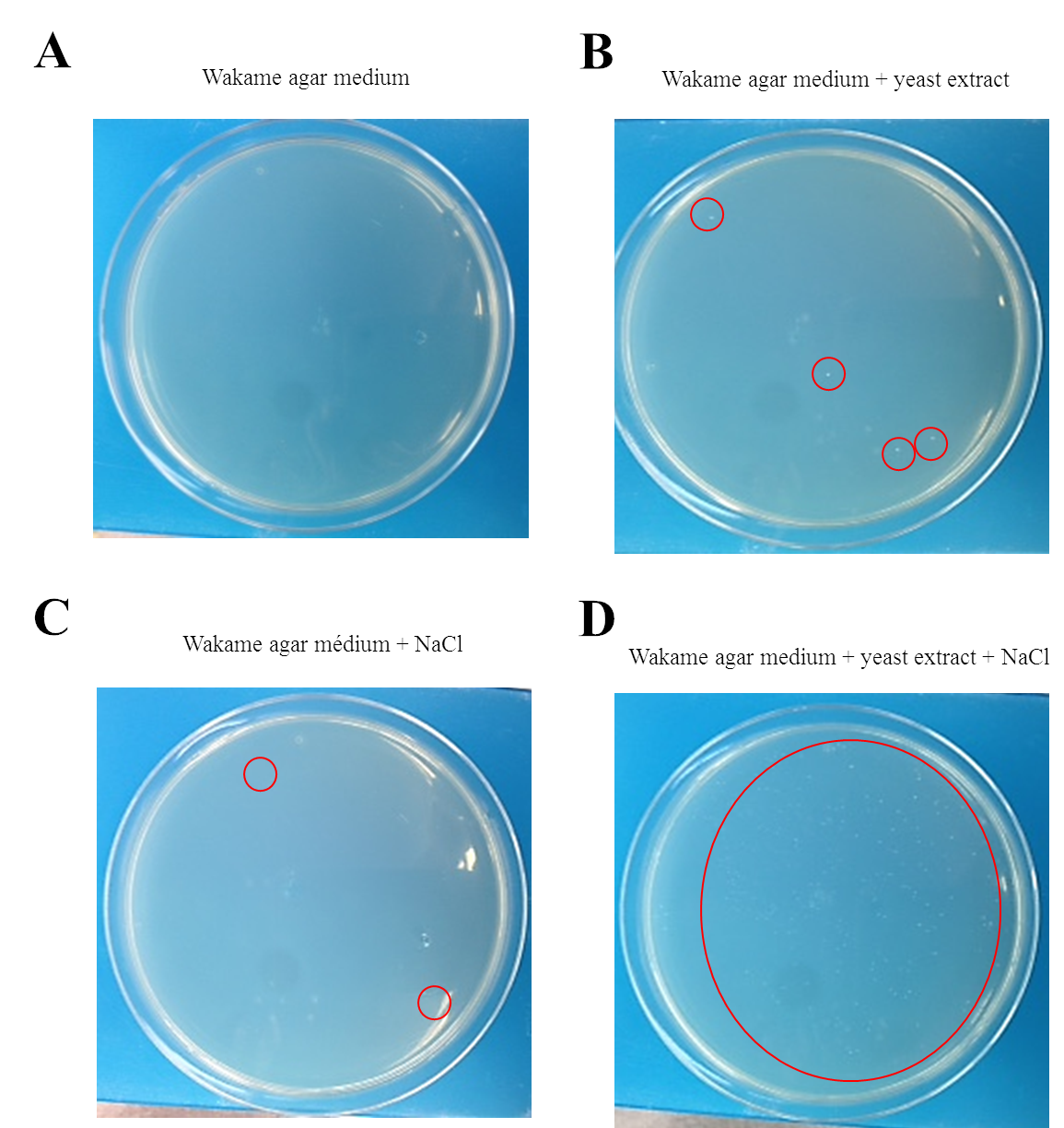

Supplement: Supplementary file 1 [file microorganisms-07-00167-s001.zip › microorganisms-470891-proofreading-supplementary/Supplementary files/Figure S1.tiff]

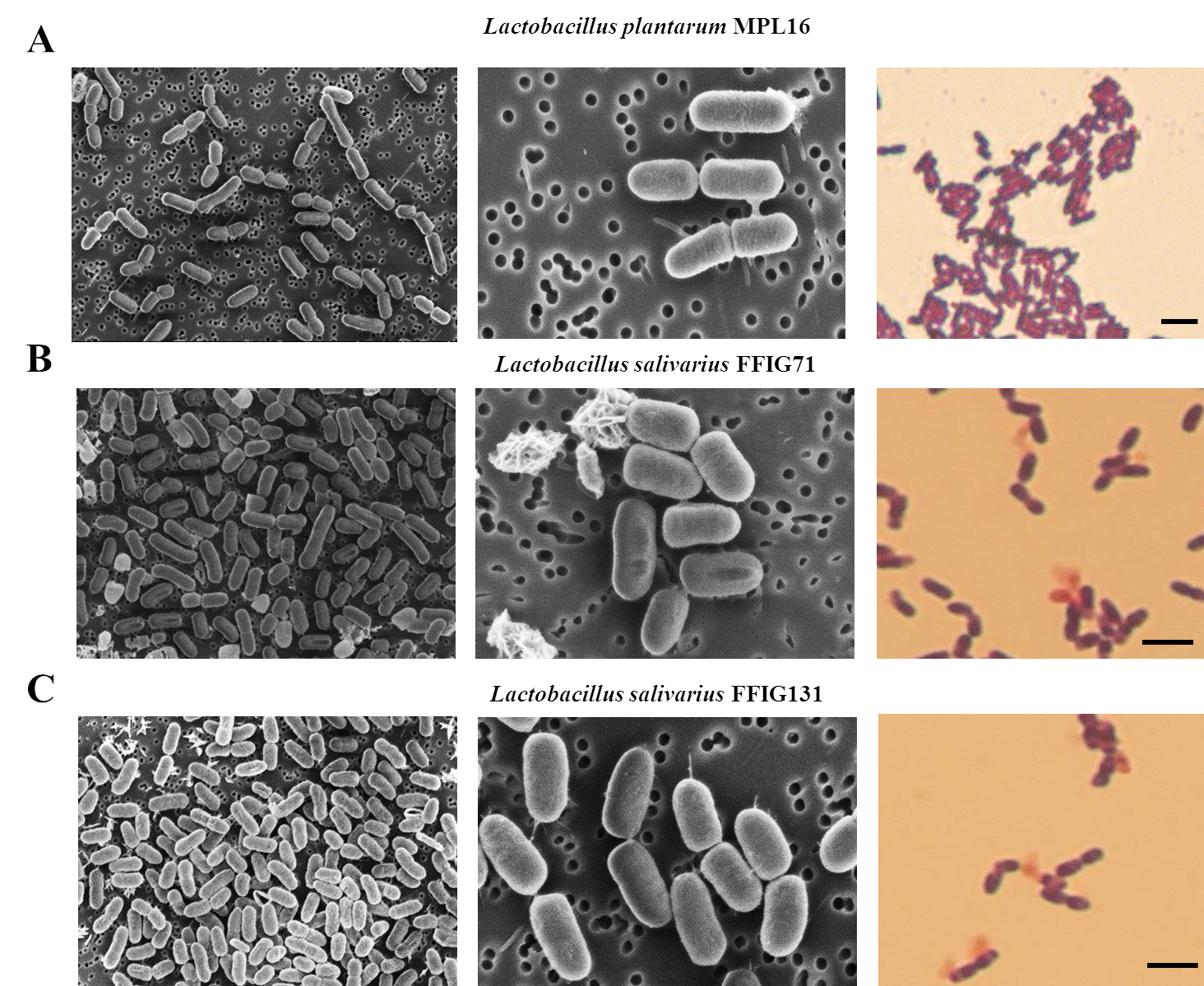

Supplement: Supplementary file 1 [file microorganisms-07-00167-s001.zip › microorganisms-470891-proofreading-supplementary/Supplementary files/Figure S2.tiff]
